# Again the Water Supply

**Published:** 1903-10

**Authors:** 


### Again the Water Supply.

AT the last meeting of the Buffalo Academy of Medicine, Dr. George E. Fell read a paper, entitled Lake and river current observations and their value to our city. Dr. Fell's conclusions were as follows:

1. Beginning at Smokes Creek,—that the lake currents follow the shore closely and pass to the United States Harbor at the first opening between the breakwaters.

2. That the lake waters, for at least one mile, out in the lake at Smokes Creek, also pass into this southerly opening. This conclusively proves that material from Smokes Creek can not pass north of first opening of breakwater.

3. That material as far out in the lake as one thousand feet from the breakwater at middle opening also passes inside or through the middle opening between breakwaters.

Also that material one thousand feet out in the lake from the red light, north end of old breakwater, passes down the river a distance of about three hundred and twenty feet easterly of our intake.

5. That material one hundred feet westerly of this red light instead of passing north of the new breakwater actually passes with a strong flow to the south of it.

6. That this last indicated current prevents any material from Smokes Creek which has reached any points inside of or to the easterly of breakwater from going to the westerly of Bird Island Pier in any quantity, if at all, and, also, the outflow of Buffalo River from passing out into the current of Niagara River as far as it did before the new breakwater was constructed, whereas it was generally believed that it produced an opposite effect.

7. It teaches us that the great mass of polluted waters from Buffalo River and Smokes Creek district, our own littoral pollution passes down the Erie Canal, instead of out or into Niagara River, except as leakage through Bird Island Pier.

8. It has settled unquestionably this fact, that the conditions existing at Buffalo are different in toto from those existing at Cleveland, Milwaukee and Chicago, and proven in the most positive manner that the location of our present intake is such that it can not be influenced detrimentally to the character of our general lake supply by local currents, or that the water at our intake is as pure as the water in the immediate center of the lake.

9. It has eliminated from the controversial probability any necessity for the construction of an enormously expensive system of filtration plants.

10. It has narrowed the question of impure water supply down to the question of possible seepage or the question of import or sedimentary disturbance from the silt at the bottom of the lake.

11. It has annihilated (I trust for good) the very general impression so extensively published to the detriment of our city that Buffalo was in great danger from its water supply; that a terrible epidemic could only be averted by boiling our water.

12. It has provided valuable data which will be of constant value in many questions arising in the future as to our water supply.

13. It has demonstrated that we are receiving water at our intake from points much farther out in the lake or even from a more satisfactory location than the water supplied to the Western New York Water Company, off Woodlawn Beach, the water of which is according to clinical observations and the investigations of Mr. George W. Fuller, now investigating our water supply, and Dr. William T. Sedgwick, of Boston, as good as any water supplied to any city in the country.

We give our readers Dr. Fell's conclusions as to the integrity of Buffalo's water supply for various reasons. The conclusions are the mature thought of a physician who has had extended experience as an engineer connected with the government works in and about our harbor. They represent the results of careful and prolonged study of the question of the purity of our water supply. They were delivered at a formal meeting of the Buffalo Academy of Medicine, and therefore in a degree carry the approval and endorsement of that distinguished body. They have appeared in the column of one of our more influential daily papers. They have received the emphatic editorial endorsement of that paper, the *Buffalo Commercial,* and they may easily be made the cause or reason for official action or inaction on the part of our Board of Health. And furthermore, we believe these same conclusions to be greatly in need of editing.

We assume at once the cordial support of our readers to the well-known proposition, that the question of our water supply is of the first importance and that the responsibility of the medical profession to guide, to teach and to inspire the public mind to right thinking, and efficient action in this regard is the chief duty of that profession. We are quite in accord with Dr. Fell, that there should be no panic as to our water supply, but we also cannot forget that for years we have had the significant fact of a mortality from typhoid fever of more than seven times that of many of the great cities of Europe, cities in which the hygenic conditions are equal to or superior to ours only in the single item of a pure water supply.

The public and those in authority must be convinced that no single fact bearing upon this question has had and will continue to have the importance of this item of clinical demonstration, of conclusive proof that the water supply of our city is contaminated with the infection of typhoid fever; proof from month to month, from year to year of the degree of the fatal activity of that infection.

The chief aim of Dr. Fell's conclusions seems to be to show the harmlessness of the water of Smokes Creek to our water supply, even assuming that water to be well loaded with the typhoid infection.

Our criticism is that his observations do not justify his conclusions, and that his conclusions are liable to be misleading and therefore dangerous, not only as to the lesser question of the possible infection of our water supply from the water of Smokes Creek, but more particularly, dangerous as to the greater question the degree of infection our water supply is receiving from the shores and bottom of Lake Erie. It is a well-known fact that the typhoid infection lives in certain soils almost indefinitely, and the inference seems safe that the mud and slime and sand of our harbor's bottom and of many spots on the near-by shores of

Lake Erie are either frequently or constantly the breeding ground of this infection and consequently a persistent menace to the health and lives of our citizens through our water supply. The frequently recurring phenomena of wind storms producing the condition of our city water, known as sand roil, must admonish the thoughtful that the shores and bottom of Lake Erie are liable to have too much influence upon the purity of our water supply, and possibly a direct causative influence upon our discreditably high typhoid morbidity and mortality.

